# Psychosis and suicide attempt following a single use of delta‐9‐tetrahydrocannabiphorol: A case report

**DOI:** 10.1002/pcn5.70060

**Published:** 2025-02-04

**Authors:** Daniel Greer, Jennifer Atherton, Justina Girgis

**Affiliations:** ^1^ Ernest Mario School of Pharmacy Rutgers University Piscataway New Jersey USA; ^2^ St. Joseph's University Medical Center Paterson New Jersey USA

**Keywords:** psychosis, substance use, suicidality, tetrahydrocannabiphorol, THCP

## Abstract

**Background:**

Delta‐9‐tetrahydrocannabiphorol (Δ^9^‐THCP) is a potent phytocannabinoid found naturally in the cannabis plant in small quantities. This cannabinoid is noted to potently bind to cannabinoid receptor 1 and cannabinoid receptor 2 and is reported to be highly psychoactive. A small amount of Δ^9^‐THCP is found naturally, which led to its isolation and synthetic production. This isolate is beginning to be sold at dispensaries. Information on Δ^9^‐THCP is sparse and case reports of adverse events add to the current literature.

**Case Presentation:**

This case report describes a patient who was brought into the emergency department with a self‐inflicted stab wound to the chest after consuming 8 mg of Δ^9^‐THCP. The patient was a regular user of standard tetrahydrocannabinol (THC) products from dispensaries and had no history of psychotic symptoms. The patient consumed the 8 mg just over 2 days prior to admission and noted psychotic symptoms for 48 h. The symptoms included depersonalization and a belief that he was already dead. A single dose of haloperidol 5 mg intravenous push was administered in the intensive care unit due to agitation. Afterwards, the patient refused all oral antipsychotics and the psychotic symptoms cleared prior to discharge.

**Conclusion:**

The case adds to the evidence suggesting that Δ^9^‐THCP is highly potent and psychoactive. Even experienced users of THC may have adverse effects to Δ^9^‐THCP due to differences in binding and potency. Isolates of phytocannabinoids require caution prior to use.

## BACKGROUND

The term “cannabinoid” refers to any chemical entity that binds to cannabinoid receptors. Common cannabinoids include delta‐9‐tetrahydrocannabinol (Δ^9^‐THC) and cannabidiol (CBD), but the *Cannabis sativa* plant contains more than 100 cannabinoids (https://www.nccih.nih.gov/health).^1^ Consumption of cannabis products is common. A survey of 387,179 Americans found that 10.1% of participants had used a cannabis product within 1 month.^2^ Despite the widespread use of cannabis products, evidence addressing the effects of different types of cannabinoids are lacking.

The cannabinoid, Δ^9^‐THC, is the main psychoactive component of the *Cannabis sativa* plant, though other cannabinoids are found naturally in the plant in varying amounts. Delta‐9‐tetrahydrocannabiphorol (Δ^9^‐THCP) is a potent and psychoactive cannabinoid that accounts for 0.0023% to 0.0136% of the total THC percentage.^3^ The alkyl side chain length of cannabinoids is thought to affect biological activity with longer chains being more active. Delta‐9‐THC has a five‐carbon alkyl side chain, while Δ^9^‐THCP has a seven‐carbon alkyl side chain. The implications of this longer side chain are substantial as Δ^9^‐THCP was found to be 33 times more active than Δ^9^‐THC at cannabinoid‐1 (CB1) receptors.^4^ The psychoactive properties of cannabinoids are related to the degree of binding to the CB1 receptors.^5^

The connection between cannabis use and psychosis is continuing to grow. Individuals with cannabis dependence are at a 3.4 times higher risk of developing a psychotic disorder. Additionally, cannabis use is associated with developing a psychotic disorder 2–3 years earlier than non‐users.^6^ Cessation of cannabis use is recommended when one experiences psychotic or paranoid symptoms.

## CASE PRESENTATION

A 41‐year‐old male was brought into the emergency department (ED) with a self‐inflicted stab wound. Two days prior to admission, the patient had consumed one alcoholic drink and 8 mg of Δ^9^‐THCP taken orally in gummy form. The patient stated that the THCP was purchased from a dispensary and noted they did not know this was different from Δ^9^‐THC. The patient noted psychotic symptoms occurring roughly 1 h after consumption, with the effects lasting for 48 h. The patient experienced depersonalization, believed that himself and his family were already dead, and believed that in order to live, he had to kill himself. The patient used a kitchen cleaver to stab himself in the chest. The patient did have a psychiatric history. He reported that 5 years previously, he had cut his wrist while intoxicated because he was feeling depressed. He started therapy at that time but noted not being consistent in seeing his provider. He denied a history of psychosis, psychiatric admissions or of seeing a psychiatrist. Additionally, he denied a family history of psychotic disorders. The patient noted regular cannabis use. The maximum amount of Δ^9^‐THC used in 1 day was 20 mg and no psychotic symptoms occurred with prior use. The patient also noted the consumption of ~20 beers per week.

Following the self‐harm attempt, a prehospital trauma activation was initiated by emergency medical services. The patient became tachypneic and hypoxic while en route to the hospital and required left‐sided needle thoracostomy. Upon arrival to the ED, the patient had an initial blood pressure of 100/60 mmHg and was noted to be pale and sleepy. He was transferred to the trauma bay and became hypotensive, with blood pressure decreasing to 80 s/40 s mmHg. A left‐sided chest tube was placed for traumatic hemothorax and the patient was transfused one unit of whole blood. Blood pressure temporarily improved to 104/71 mmHg following chest tube placement and blood transfusion; however, dropped to 93/46 mmHg with a mean arterial pressure of 62, at which point norepinephrine was started. Once stabilized, the patient was immediately transferred to the operating room for a left anterior thoracotomy, left upper lobe wedge resection and washout, and closure of the self‐inflicted stab wound, during which the patient lost an estimated 1.5 L of blood.

The patient was then transferred to the surgical intensive care unit (SICU) off of any pressure support or sedation. While in the SICU, he became uncooperative with examinations and began to make inappropriate religious remarks, attempted to get out of bed, and forcefully removed his Foley catheter and chest tubes. The patient was noted to have a flat affect, and began to ignore questions. At this point, an electrocardiogram was completed and showed a QTc of 419. The patient was given a single dose 5 mg of haloperidol intravenous (IV) push, and a behavioral health consult was placed.

The consult psychiatrist recommended adding lurasidone 20 mg daily with food to address mood and psychosis as well as 5 mg of haloperidol IV push every 6 h as needed for agitation. Lurasidone was then switched to aripiprazole 5 mg daily given formulary restrictions, though the patient refused all doses of aripiprazole. The mental status exam noted some confusion, a flat affect, and impaired insight and judgement.

During the patient's second day, he remained off of vasopressors and the anterior chest tube was removed. The patient was noted to have left upper extremity swelling on the third day in the SICU. A venous Doppler found an occlusive thrombus in the distal brachial vein, for which a heparin infusion was initiated. Behavioral health recommended adding hydroxyzine pamoate 25 mg every 6 h for anxiety. The mental status exam noted a constricted affect, and poor judgment, but stated the patient had linear thought processes, euthymic mood, and was calm. The patient's posterior chest tube was also removed.

On Day 4, the patient was transitioned to enoxaparin for the management of the upper extremity deep vein thrombosis at a dose of 1 mg/kg twice daily. The patient was medically cleared by trauma surgery for transfer to inpatient psychiatry for further crisis evaluation.

On Day 7 of admission, the patient was transferred to inpatient psychiatry with a psychiatric diagnosis of cannabis‐induced psychotic disorder. The patient refused aripiprazole 5 mg daily. After being seen by the psychiatrist, the medication was discontinued, as the patient denied residual psychotic or psychiatric symptoms. The mental status exam contained no abnormalities at this time. The patient was pleasant and an active participant in the unit. A family meeting was held, the patient agreed to go to inpatient substance use rehabilitation and was discharged to the rehabilitation facility on Day 12 of admission.

## DISCUSSION

Many types of phytocannabinoids have been detected in the cannabis plant but few have been isolated and characterized. Potential benefits and risks may emerge as more isolates of phytocannabinoids are individually studied because isolated phytocannabinoids may have differing degrees of psychoactive properties.

The potency, frequency, and duration of cannabis use are all associated with first‐episode psychosis.^7^ One study found that 0.47% of people who use cannabis report cannabis induced psychotic symptoms. The risk is particularly elevated in younger individuals, those who used products with higher potency, and those with a previous diagnosis of psychosis, bipolar disorder, anxiety, or depression.^8^ Case reports support these claims as well. For example, a 2020 case report highlights a patient who presented to the emergency department with paranoid delusions after using a highly potent formulation of cannabis containing up to 80% THC.^9^ The consequences of cannabis‐induced psychosis can be severe as seen with the self‐inflicted stab wound that occurred in our patient. Additional reports call attention to the severity. A case report highlighted a 23‐year‐old patient who smoked 2 g of cannabis and then used scissors to self‐amputate his penis.^10^ The review of existing literature also emphasizes the age difference between typical cases and our patient, as most studies concentrate on substance use during adolescence, which is linked to an increased risk of psychosis when the person is in their twenties.^11^ In contrast, our patient presented at the age of 41. The treatment of cannabis‐induced psychosis is not fully understood and the patient in this case had his psychosis resolved after only receiving one dose of haloperidol 5 mg.

The dosing and route of administration of cannabinoids are still being investigated, with higher doses being associated with more adverse reactions such as psychosis or paranoia. For chronic pain management, a dosing protocol typically initiates treatment with 2.5–5 mg of THC, increases by 2.5–5 mg THC every 2–3 days, and recommends a maximum dose of 40 mg THC daily.^12^ A review of insomnia trials concluded that 10–30 mg of THC showed positive effects but also noted that the 30 mg dose was poorly tolerated.^13^ Another review article also concluded that the general maximum dose of medical THC should be 30 mg daily, preferably administered with CBD.^14^ However, the optimal dosing of THC has not been fully established and there is even more limited information on the dosing of Δ^9^‐THCP. This lack of dosing guidance may place patients at risk because the effects from THC do not equate to the effects from Δ^9^‐THCP. Additionally, the route of administration may alter the effects of the cannabinoid. Limited information is available for different routes of Δ^9^‐THCP, but differences are being seen based on route of administration for Δ^9^‐THC.^15^

The relationship between mental illness, substance use disorders, and suicidality is complex. A survey of 281,650 adults found that cannabis use was associated with higher prevalence of suicidal ideations (adjusted risk ratio [ARR] 1.4), plans (ARR 1.6), and attempts (ARR 1.4). The survey also found that individuals with cannabis use disorder, daily users without a use disorder, and non‐daily users of cannabis were all at an increased risk of suicidal ideation, plans, and attempts.^16^ Younger populations may be affected more than other populations. A trial analyzing the effect of marijuana legalization in Washington and Colorado found that suicides among those aged 15–24 years increased by 17.9% following legalization in Washington.^17^

## CONCLUSION

This case highlights the importance of isolating and studying individual phytocannabinoids. Those who use cannabis may be unaware of the potency and varying effects of different cannabinoids, especially as new isolates become available. Additionally, the intersection of mental illness, use disorders, and suicidality should be discussed with at‐risk patients.

## AUTHOR CONTRIBUTIONS

Daniel Greer conceptualized the idea for this case report. All authors provided critical review and revision of the manuscript. All authors provided care for the case study patient and drafted the initial case report manuscript. All authors approved the final manuscript as submitted and agree to be accountable for all aspects of the work.

## CONFLICT OF INTEREST STATEMENT

The authors declare no conflict of interest.

## ETHICS APPROVAL STATEMENT

Informed consent was obtained from the patient and the institution agreed to the publication.

## PATIENT CONSENT STATEMENT

The patient provided written informed consent for publication.

## CLINICAL TRIAL REGISTRATION

N/A

## Data Availability

The data of this case report are available from the corresponding author upon reasonable request.

## References

[1] National Center for Complementary and Integrative Health . Cannabis (Marijuana) and Cannabinoids: What You Need To Know. https://www.nccih.nih.gov/health/cannabis‐marijuana‐and‐cannabinoids‐what‐you‐need‐to‐know#:~:text=How%20many%20cannabinoids%20are%20there,other%20cannabinoids%20have%20been%20identified. Accessed 1 Oct 2024.

[2] Jeffers AM , Glantz S , Byers A , Keyhani S . Sociodemographic characteristics associated with and prevalence and frequency of cannabis use among adults in the US. JAMA Netw Open. 2021;4(11):e2136571. Epub 2021 Nov 1. 10.1001/jamanetworkopen.2021.36571 34846523 PMC8634054

[3] Bueno J , Greenbaum EA . (−)‐trans‐Δ^9^‐Tetrahydrocannabiphorol content of *Cannabis sativa* inflorescence from various chemotypes. J Nat Prod. 2021;84(2):531–536. 10.1021/acs.jnatprod.0c01034 33565878

[4] Citti C , Linciano P , Russo F , Luongo L , Iannotta M , Maione S , et al. A novel phytocannabinoid isolated from *Cannabis sativa* L. with an in vivo cannabimimetic activity higher than Δ^9^‐tetrahydrocannabinol: Δ^9^‐tetrahydrocannabiphorol. Sci Rep. 2019;9(1):20335. Epub 2019 Dec 30. 10.1038/s41598-019-56785-1 31889124 PMC6937300

[5] Little R , D'mello D . A cannabinoid hypothesis of Schizophrenia: pathways to psychosis. Innov Clin Neurosci. 2022;19(7–9):38–43. 36204167 PMC9507146

[6] Hasan A , von Keller R , Friemel CM , Hall W , Schneider M , Koethe D , et al. Cannabis use and psychosis: a review of reviews. Eur Arch Psychiatry Clin Neurosci. 2020;270(4):403–412. 10.1007/s00406-019-01068-z 31563981

[7] Di Forti M , Morgan C , Dazzan P , Pariante C , Mondelli V , Marques TR , et al. High‐potency cannabis and the risk of psychosis. Br J Psychiatry. 2009;195(6):488–491. 10.1192/bjp.bp.109.064220 19949195 PMC2801827

[8] Schoeler T , Ferris J , Winstock AR . Rates and correlates of cannabis‐associated psychotic symptoms in over 230,000 people who use cannabis. Transl Psychiatry. 2022;12(1):369. Epub 2022 Sep 6. 10.1038/s41398-022-02112-8 36068202 PMC9448725

[9] Rossi G , Beck M . A little dab will do: a case of cannabis‐induced psychosis. Cureus. 2020;12(9):e10311. Epub 2020 Sep 8. 10.7759/cureus.10311 33052273 PMC7544610

[10] Jengsuebsant N , Benjachaya S , Vuthiwong J , Tangsuwanaruk T . Penile self‐amputation due to cannabis‐induced psychosis: a case report. J Med Case Reports. 2022;16(1):37. Epub 2022 Jan 30. 10.1186/s13256-022-03267-0 PMC8801084 35093161

[11] Hall W , Degenhardt L . Cannabis use and the risk of developing a psychotic disorder. World Psychiatry. 2008;7(2):68–71. 10.1002/j.2051-5545.2008.tb00158.x 18560513 PMC2424288

[12] Bhaskar A , Bell A , Boivin M , Briques W , Brown M , Clarke H , et al. Consensus recommendations on dosing and administration of medical cannabis to treat chronic pain: results of a modified Delphi process. J Cannabis Res. 2021;3(1):22. Epub 2021 Jul 2. 10.1186/s42238-021-00073-1 34215346 PMC8252988

[13] Velzeboer R , Malas A , Boerkoel P , et al. Cannabis dosing and administration for sleep: a systematic review [published correction appears in Sleep. 2023 Mar 9;46(3):zsad008. 10.1093/sleep/zsad008]. Sleep. 2022;45(11):zsac218 36107800

[14] MacCallum CA , Russo EB . Practical considerations in medical cannabis administration and dosing. Eur J Intern Med. 2018;49:12–19. 10.1016/j.ejim.2018.01.004 29307505

[15] Hart C , Ward A , Haney M , Comer S , Foltin R , Fischman M . Comparison of smoked marijuana and oral Δ^9^‐tetrahydrocannabinol in humans. Psychopharmacology. 2002;164(4):407–415. 10.1007/s00213-002-1231-y 12457271

[16] Han B , Compton WM , Einstein EB , Volkow ND . Associations of suicidality trends with cannabis use as a function of sex and depression status. JAMA Netw Open. 2021;4(6):e2113025. Epub 2021 Jun 1. 10.1001/jamanetworkopen.2021.13025 34156452 PMC8220498

[17] Doucette ML , Borrup KT , Lapidus G , Whitehill JM , McCourt AD , Crifasi CK . Effect of Washington State and Colorado's cannabis legalization on death by suicides. Prev Med. 2021;148:106548. 10.1016/j.ypmed.2021.106548 33838156

